# Molecular Cytogenetics of Eurasian Species of the Genus *Hedysarum* L. (Fabaceae)

**DOI:** 10.3390/plants10010089

**Published:** 2021-01-04

**Authors:** Olga Yu. Yurkevich, Tatiana E. Samatadze, Inessa Yu. Selyutina, Svetlana I. Romashkina, Svyatoslav A. Zoshchuk, Alexandra V. Amosova, Olga V. Muravenko

**Affiliations:** 1 Engelhardt Institute of Molecular Biology, Russian Academy of Sciences, 32 Vavilov St, 119991 Moscow, Russia; olikys@gmail.com (O.Y.Y.); tsamatadze@gmail.com (T.E.S.); slavazo@mail.ru (S.A.Z.); amomar@mail.ru (A.V.A.); 2 Central Siberian Botanical Garden, SB Russian Academy of Sciences, 101 Zolotodolinskaya St, 630090 Novosibirsk, Russia; selyutina.inessa@mail.ru; 3 All-Russian Institute of Medicinal and Aromatic Plants, 7 Green St, 117216 Moscow, Russia; Romashkin69@inbox.ru

**Keywords:** genus *Hedysarum*, species distribution areas, chromosome variability, minor 45S rDNA sites

## Abstract

The systematic knowledge on the genus *Hedysarum* L. (Fabaceae: Hedysareae) is still incomplete. The species from the section *Hedysarum* are valuable forage and medicinal resources. For eight *Hedysarum* species, we constructed the integrated schematic map of their distribution within Eurasia based on currently available scattered data. For the first time, we performed cytogenomic characterization of twenty accessions covering eight species for evaluating genomic diversity and relationships within the section *Hedysarum*. Based on the intra- and interspecific variability of chromosomes bearing 45S and 5S rDNA clusters, four main karyotype groups were detected in the studied accessions: (1) *H.* *arcticum*, *H. austrosibiricum*, *H. flavescens*, *H. hedysaroides*, and *H. theinum* (one chromosome pair with 45S rDNA and one pair bearing 5S rDNA); (2) *H. alpinum* and one accession of *H. hedysaroides* (one chromosome pair with 45S rDNA and two pairs bearing 5S rDNA); (3) *H. caucasicum* (one chromosome pair with 45S rDNA and one chromosome pair bearing 5S rDNA and 45S rDNA); (4) *H. neglectum* (two pairs with 45S rDNA and one pair bearing 5S rDNA). The species-specific chromosomal markers detected in karyotypes of *H. alpinum, H. caucasicum*, and *H. neglectum* can be useful in taxonomic studies of this section.

## 1. Introduction

The genus *Hedysarum* L. (Fabaceae: Hedysareae) comprises about 200 species of annual or perennial herbs distributed in Asia, Europe, North Africa, and North America which adapt to diverse habitats including temperate forests, stepped, polar and high-mountain regions [1,2,3].

The systematic knowledge on the genus *Hedysarum* is still incomplete, with uncertainty in generic delimitation. Fairly recently, the most prevalent Fedtschenko’s classification [1], which divided *Hedysarum* into seven sections according to their habits and morphology of stems and loments, was revised by Choi and Ohashi [4]. Currently, based on comprehensive morphological studies, the section *Gamotion* [5] is renamed into the sect. *Hedysarum* [4]. However, within the sections of the genus, the presence of morphologically intermediate forms and also significant intraspecific morphological diversity complicate discrimination between closely related species [2,6]. Moreover, only scattered data on the species occurrence in Eurasia are currently available, and spatial overlap in species distributions is still uncertain.

There are some differences in carpological and anatomical features of fruits between the species from the sect. *Hedysarum* (=syn. *Gamotion*) and other sections of the genus *Hedysarum* [7]. Also, the medicinal species from this section contain different bioactive compounds in leaves and roots, which are responsible for their immunomodulatory, antioxidant, anti-tumor and anti-diabetic effects [8,9,10]. In particular, several species, *H. neglectum* Ledeb., *H. alpinum* L., *H. theinum* Krasnob., and *H. flavescens* Rgl. et Schmalh., are rich in xanthone magniferine and oligomeric catechins, which makes them valuable raw material for the production of multifunctional biologically active additives, and thus offers the opportunity to develop new effective herbal remedies, in particular, a multifunctional antiviral drug ‘Alpisarin’ [10,11,12,13,14,15]. Some species from the section *Hedysarum*, such as *H. flavescense*, *H. neglectum* and *H. alpinum*, are valuable forage resources in their distribution areas [16,17,18].

At the same time, natural resources of *Hedysarum* species are insufficient for ever-growing needs. In view of this, *H. alpinum* and *H. theinum* are already being cultivated and/or introduced into the cell culture by means of biotechnological techniques [19,20]. However, such approaches need comprehensive comparative studies of genetic traits in valuable species and their closest relatives. In the genus *Hedysarum*, karyological studies were mostly performed by simple monochrome staining of chromosomes [21,22,23] or C-banding [24]. As reported earlier, the species belonging to the sect. *Hedysarum* have the basic chromosome number x = 7 unlike the species from other sections having x = 8 [4,21,25]. FISH-based chromosomal localization of rRNA genes was carried out in five Algerian species [26,27]. Molecular cytogenetic analysis of other *Hedysarum* species has not been conducted, and intra- or interspecific variability in chromosome localization of rRNA genes still remains uncertain.

Currently, taxonomic and phylogenetic relationships within the genus *Hedysarum* are being studied intensively with the use of various molecular phylogenetic approaches [28,29,30,31,32,33,34,35]. Recent extensive phylogenetic studies of Hedysareae based on nuclear (ITS, ETS, PGDH, SQD1, TRPT) and plastid (psbA-trnH, trnC-petN, trnL-trnF, trnS-trnG, petN-psbM) DNA sequencing did not support the monophyly of the genus *Hedysarum* [33,34,35]. Duan et al. [33], using nrDNA ITS and three plastid regions (*mat*K, *trn*L–F, *trn*H–*psb*A), recognized three clades within the genus *Hedysarum* corresponding to the sections *Hedysarum*, *Multicaulia*, and *Stracheya*. Later, however, the incongruent position of the *Hedysarum s*.*s*. clade between the nuclear and plastid trees was revealed that could be related to a chloroplast capture hypothesis via introgression [34]. The sect. *Stracheya* clade was resolved as sister to the sect. *Hedysarum* clade in both nuclear and plastid trees, which supports merging *Stracheya* into *Hedysarum* [34]. These findings are consistent with the differentiation of the genus *Hedysarum* into three sections, with the species grouped according to their basic chromosome numbers x = 7 (the section *Hedysarum*) or x = 8 (the sections *Multicaulia* and *Stracheya*) [4,21,25]. At the same time, within the sect. *Hedysarum*, comparative analyses of nuclear and plastid genome sequences have been performed only in few species, and genomes of a number closely related medicinal species distributed in Eurasia still remain unstudied [33,34,35], and comprehensive investigation of cytogenetic peculiarities is essential for clarifying their taxonomy and phylogenetic relationships.

In order to characterize intra- and interspecific genomic diversity and also evaluate relationships within the genus *Hedysarum* sect. *Hedysarum*, we performed molecular cytogenetic characterization of twenty Eurasian accessions covering eight closely related species. Additionally, spatial overlap in species distributions within Eurasia was analyzed and the integrated schematic map of the species areas was constructed.

## 2. Results

### 2.1. Distribution Areas of the Studied Hedysarum Species

**Figure 1:**
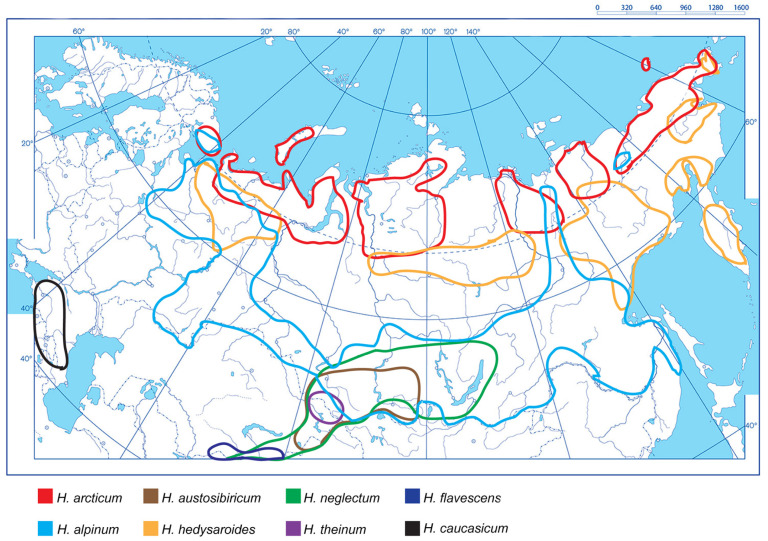
An integrated schematic map showing distribution of *H. alpinum*, *H. arcticum*, *H. austrosibiricum*, *H. caucasicum*, *H. flavescens*, *H. hedysaroides*, *H. neglectum* and *H. theinum* within the northern, central and eastern parts of Eurasia. The species names and correspondent colours of the lines indicating the boundaries of the species occurrence are specified under the maps.

For *H. alpinum*, *H. hedysaroides* (L.) Schinz et Thell., *H. arcticum* B. Fedtsch., *H. austrosibiricum* B. Fedtsch., *H. neglectum*, *H. theinum*, *H. flavescens*, and *H. caucasicum* M. Bieb., we constructed an integrated schematic map of their distribution in the northern, central and eastern parts of Eurasia based on the analysis of the currently available data [6,16,17,18,36,37,38,39,40] (Figure 1).

In these regions, *H. alpinum* occurs in Kola Peninsula and North-Eastern Europe and also in Siberia, Kamchatka and Far East regions. *H. arcticum* is widespread in the Arctic and Sub-Arctic regions of the Eurasian continent from Arctic Scandinavia and Kola Peninsula to Chukotka Peninsula [6,16,36]. *H. hedysaroides* is distributed in the North-Eastern Europe, Central Siberia and the Far East regions [38].

*H. austrosibiricum* is an endemic species which normally occurs in the highlands of Southern Siberia (the Altai Mountains, Kuznetskii Alatau and the eastern edge of the Sayan Mountains) [37]. *H. theinum* is a highland alpine endemic species which grows in the Western Altai Mountains and also in the mountain ranges of Western Mongolia at altitudes of 1300–2000 m [36]. *H. neglectum* is distributed in the Altai Mountains, Kuznetskii Alatau, Western and Eastern Sayan Mountains and South-western parts of Tuva at lower altitudes (500–1500 m) compared to *H. theinum* [36]. *H. flavescens* occupies a narrow ecological niche growing on the limited small areas within the mountain ranges of the Western Tian Shan, Pamir-Alay Mountains [17,18]. This species normally occurs at altitudes of 2500–3100 m and less frequently, at medium altitudes (1000–2500 m) [17,18]. *H. caucasicum* is a highland species distributed in the Caucasus, Republic of Adygea and Krasnodar region at altitudes of 1500–3000 m [16,39,40].

**Figure 2:**
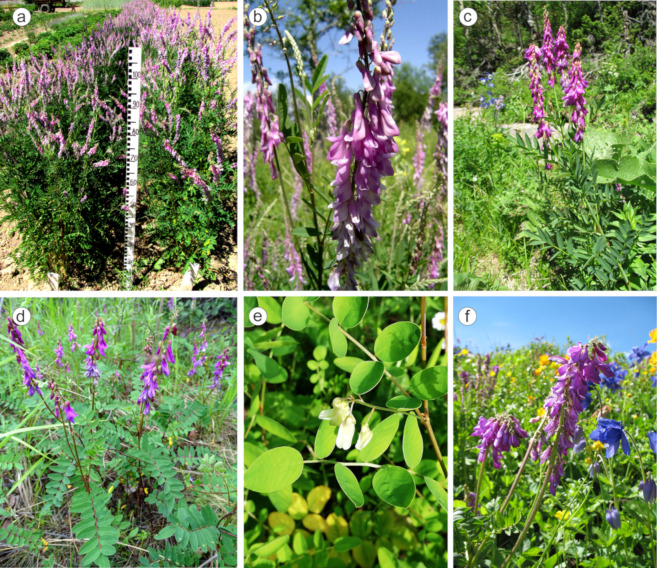
*H. alpinum* plants growing on the trial plot (AIMAP, Moscow) (**a**) and wild populations of *H. alpinum* (the Zabaikalye region) (**b**), *H. theinum* (the Altai Mountains) (**c**), *H. hedysaroides* (the Magadan region) (**d**), *H. flavescens* (AIMAP, Moscow) (**e**) and *H. neglectum* (the Altai region) (**f**). The photos were taken by S.I. Romashkina (**a**,**e**) and I.Yu. Selyutina (**b**–**d**,**f**).

The constructed schematic map demonstrates ecological diversity in the species occurrences and also indicates the regions where several *Hedysarum* species grow together. The areas of *H. alpinum*, *H. hedysaroides,* and *H. arcticum* overlap partly, and the ranges of *H. austrosibiricum*, *H. neglectum*, *H. theinum* and *H. alpinum* overlap almost totally. Moreover, *H. flavescens* and *H. neglectum* have similar geographical areas within the mountain ranges of the Pamir and Altai Mountains. The distribution area of *H. caucasicum* confines to the Caucasus region and has no areas overlapping with the other studied species (Figure 1). In Figure 2, several studied *Hedysarum* species growing on a trial plot or in their natural habit are presented.

### 2.2. Chromosomal Structural Variations in the Studied Species

**Figure 3:**
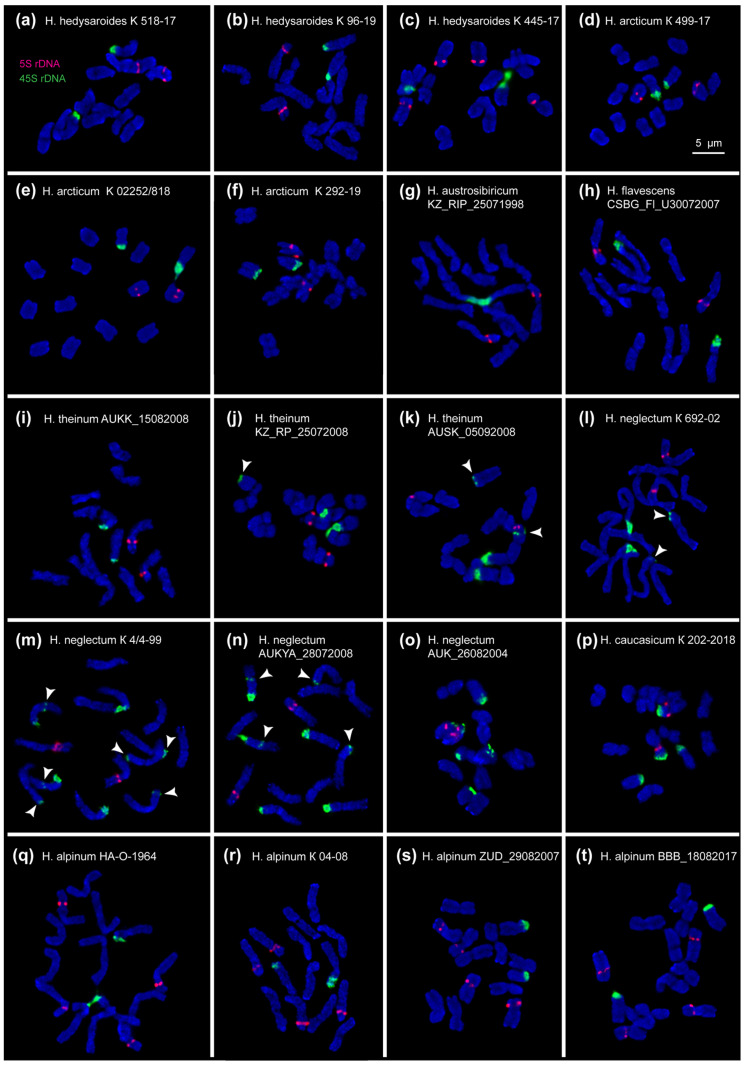
Metaphase plates of the studied accessions of *H. hedysaroides* (**a**–**c**), *H. arcticum* (**d**–**f**), *H. austrosibiricum* (**g**), *H. flavescens* (**h**), *H. theinum* (**i**–**k**), *H. neglectum* (**l**–**o**), *H. caucasicum* (**p**) and *H. alpinum* (**q**–**t**) after FISH with 45S rDNA (green) and 5S rDNA (red). DAPI chromosome staining—blue. Arrowheads point to polymorphic sites of 45S rDNA. Bar—5 μm.

FISH mapping of 45S and 5S rDNA clusters on chromosomes of the studied *Hedysarum* species was performed for the first time (Figure 3).

**Figure 4:**
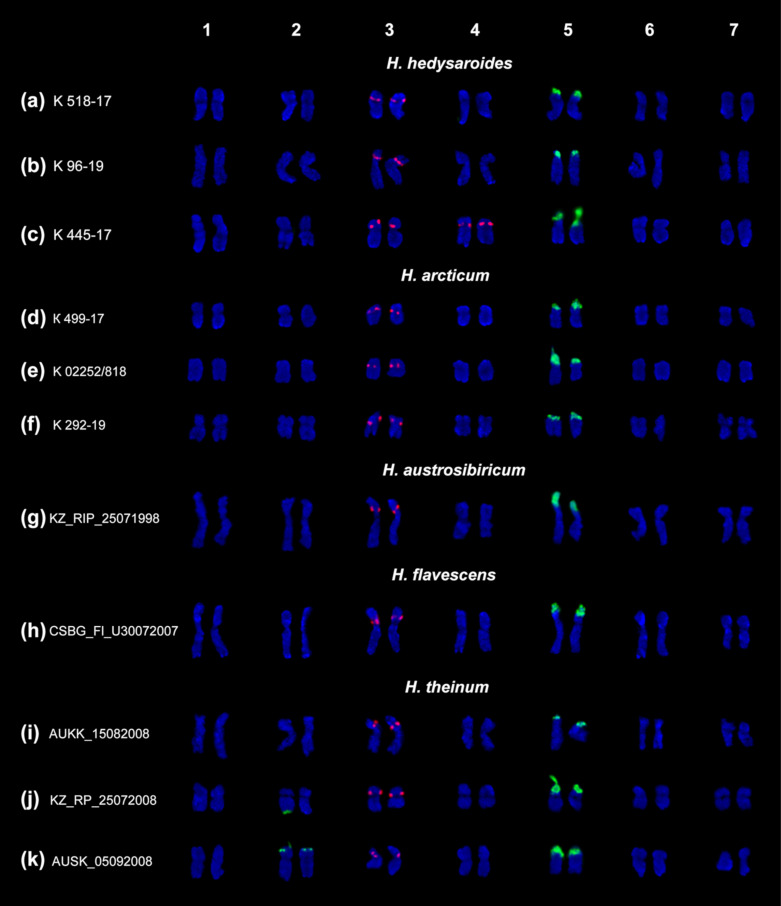
Karyotypes of the studied accessions of *H. hedysaroides* (**a**–**c**), *H. arcticum* (**d**–**f**), *H. austrosibiricum* (**g**), *H. flavescens* (**h**) and *H. theinum* (**i**–**k**). Karyograms of the metaphase plates shown in Figure 3 after FISH with 45S rDNA (green) and 5S rDNA (red). DAPI chromosome staining—blue.

**Figure 5:**
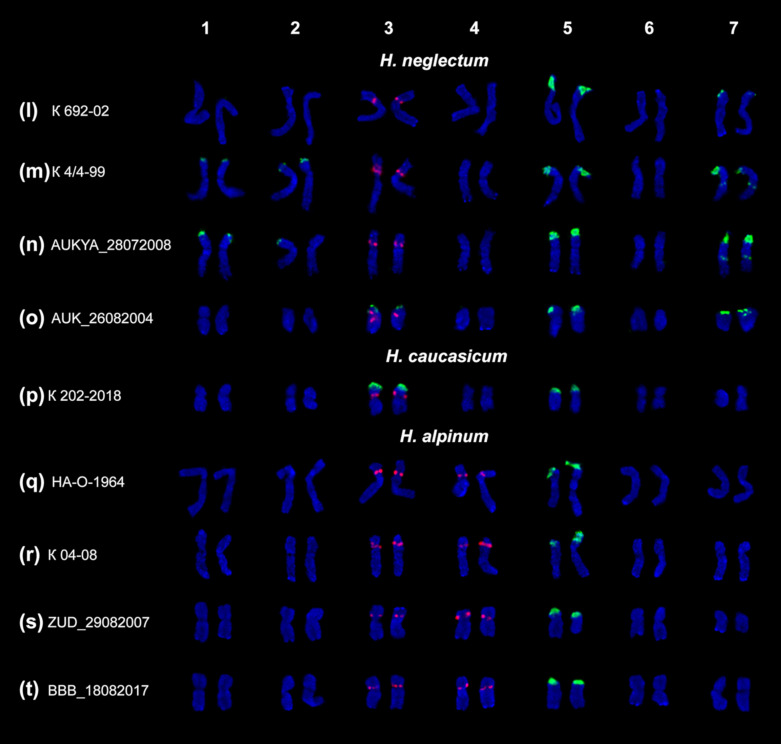
Karyotypes of the studied accessions of *H. neglectum* (**l**–**o**) and *H. caucasicum* (**p**) and *H. alpinum* (**q**–**t**). Karyograms of the metaphase plates shown in Figure 3 after FISH with 45S rDNA (green) and 5S rDNA (red). DAPI chromosome staining—blue.

Based on chromosomal morphology, DAPI-banding patterns and distribution of 45S and 5S rDNA clusters, the species karyograms were constructed (Figure 4 and Figure 5).

All studied accessions have diploid karyotypes with 2n = 14 chromosomes. In *H. flavescens* and *H. caucasicum*, the chromosome numbers were determined for the first time. The examined karyotypes involved metacentric and submetacentric chromosomes (Figure 4h and Figure 5p).

In karyotypes of *H. hedysaroides*, *H. arcticum*, *H. austrosibiricum*, *H. theinum* and *H. flavescens* we observed one similar pair of major clusters of each 45S and 5S rDNA (Figure 4). In *H. theinum*, we also detected polymorphic minor 45S rDNA sites but only in chromosome pair 2 (in heteromorphic or homomorphic combinations) (Figure 4j,k).

Karyotypes of the examined accessions of *H. neglectum* and *H. caucasicum* included two pairs of satellite chromosomes (Figure 5l–p). The second major 45S rDNA cluster was localized in the short arms of chromosome pairs 3 (in *H. caucasicum*) and 7 (in *H. neglectum*). Also, in karyotypes of *H. neglectum*, polymorphic minor 45S rDNA sites were revealed in chromosome pairs 1, 2, 3 or 7 (Figure 5m–o).

In karyotypes of *H. alpinum* (Figure 5q–t) and one accession of *H. hedysaroides* (K 445-17) (Figure 4c), we observed two chromosome pars bearing major 5S rDNA clusters, without considerable changes in morphology of these chromosomes.

However, the chromosomal localization of rDNA clusters observed in the studied species, was not uniform, and polymorphic variants (in different combinations or independently) were revealed among individuals of the same accession as well as among different accessions.

Variant NOR-I was observed in *H. austrosibiricum* and *H. flavescence*. A satellite chromosome pair (5) was presented as a medium-sized submetacentric chromosome with a large satellite localized in the short arms. A 45S rDNA cluster was localized on the satellite and adjacent chromosome region (Figure 4g,h).

NOR-II was revealed in *H. hedysaroides*, *H. arcticum* and *H. theinum*. A satellite chromosome pair (5) was presented as a small-sized submetacentric chromosome with a 45S rDNA cluster localized in the distal region, and a small satellite was sometimes dispersed into a cloud (Figure 4a–f,i–k).

In karyotypes of *H. alpinum* (Figure 5q–t) and one accession of *H. hedysaroides* (K 445-17) (Figure 4c), we observed both variants (NOR-I and NOR-II) in homomorphic and/or heteromorphic combinations.

Variant NOR-negl denotes chromosome pair 7 with a 45S rDNA cluster localized in the distal region and a small satellite, which was often dispersed and poorly stained with DAPI. NOR-negl was revealed in karyotypes of *H. neglectum* together with NOR-I (chromosome pair 5) (Figure 5l–o). Additionally, in *H. neglectum* accessions K 4/4-99 and AUKYA_28072008, we detected minor polymorphic sites of 45S rDNA localized in the median regions of the long arms of chromosome pair 7 (Figure 5m,n).NOR-cauc denotes a pair of chromosomes (3) with a small dispersed satellite bearing a 45S rDNA cluster (localized in the distal region) and also a 5S rDNA cluster (revealed in the pericentromeric region of the short arms). Both NOR-cauc and NOR-II (chromosome 5) variants were detected in karyotypes of *H. caucasicum* (Figure 5p).

In *H. neglectum* (accessions K 4/4-99 and AUKYA_28072008), minor sites 45S rDNA were revealed in the distal regions of the short arms of chromosome pairs 1 and 2 (in homomorphic and/or heteromorphic variants) (Figure 5m,n). In *H. theinum*, minor 45S rDNA clusters were revealed in the distal regions of the short (accession AUSK_05092008; homomorphic variant) (Figure 4k) and the long (accession KZ_RP_25072008; heteromorphic variant) (Figure 4j) arms of chromosome pair 2.

Besides, we revealed several variants of localization of 5S rDNA clusters.

A similar variant 5S-sim was observed in all examined accessions. A 5S rDNA cluster was localized in the proximal region of the short arms of chromosome pair 3 (Figure 4 and Figure 5). In two accessions of *H. neglectum*, K 692-02 (in three plants) and AUK_26082004 (in all studied plants), variant 5S-negl was detected. On chromosome pair 3, we observed a major 5S rDNA cluster localized similar to variant 5S-sim, and also a minor 45S rDNA site revealed in the distal region of the short arms (Figure 5o).

Besides, in karyotypes of all studied accessions of *H. alpinum* (Figure 5q–t) and one accession of *H. hedysaroides* (K 445-17) (Figure 4c), we revealed variant 5S-sim (chromosome 3) together with variant 5S-alp which denoted a 5S rDNA cluster localized in the proximal region of the short arms of chromosome pairs 4.

**Figure 6:**
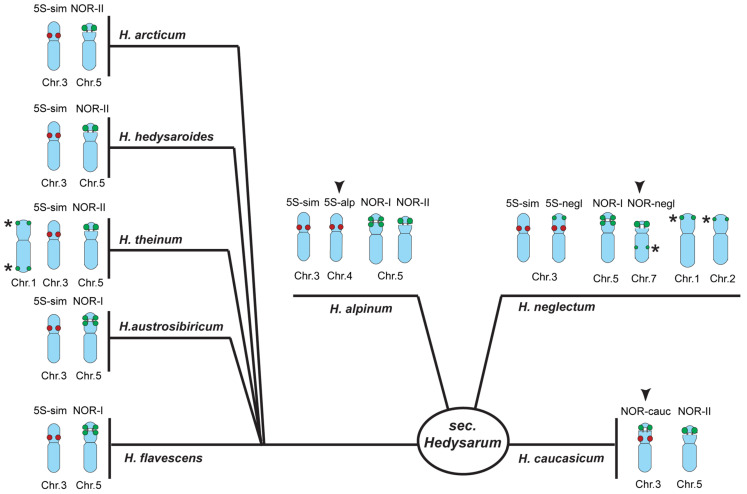
Four main groups of karyotypes within the sect. *Hedysarum* based on chromosome localization of 45S rDNA and 5S rDNA clusters. Heads of arrows indicate the revealed species-specific chromosome markers. Asterisks denote polymorphic minor 45S rDNA sites.

Based on chromosomal localization of 45S and 5S rDNA clusters, we divided all studied species from the sect. *Hedysarum* into four main karyotypic groups: (1) *H. arcticum*, *H. austrosibiricum*, *H. flavescens*, *H. hedysaroides* and *H. theinum*; (2) *H. alpinum* and one accession K 445-17 of *H. hedysaroides*; (3) *H. caucasicum* and (4) *H. neglectum* (Figure 6).

## 3. Discussion

Previous phylogenetic data indicated non-monophyly of the genus *Hedysarum* in relation to *Onobrychis*, *Greuteria*, *Eversmannia*, *Corethrodendron*, *Taverniera*, and *Ebenus*. At the same time, both the cp and nr trees resolved the sect. *Hedysarum* as a monophyletic clade [33,35]. The species of the sect. *Hedysarum* have the basic chromosome number x = 7 in contrast to the other sections of the genus *Hedysarum* having the basic chromosome number x = 8 [4,21,25,36]. Karyotypes of the examined here accessions of *H. alpinum*, *H. arcticum*, *H. austrosibiricum*, *H. caucasicum*, *H. flavescens*, *H. hedysaroides*, *H. neglectum*, and *H. theinum* also comprise seven chromosome pairs. Moreover, we did not reveal any tetraploid karyotypes among the studied accessions though tetraploids were described earlier for these species [25,36,41]. In the present study, all examined species from the sect. *Hedysarum* were divided into four main groups according to the chromosome localization of 45S and 5S rDNA clusters: (1) *H. arcticum*, *H. austrosibiricum*, *H. flavescens*, *H. hedysaroides* and *H. theinum*; (2) *H. alpinum* and one accession K 445-17 of *H. hedysaroides*; (3) *H. caucasicum* and (4) *H. neglectum*. In karyotypes of all studied species we observed similar distribution of major 5S and 45S rDNA clusters on chromosome pairs 3 and 5, correspondingly that might suggest their close relationship. These similarities in distribution of major 45S and 5S rDNA clusters are consistent with the monophyletic origin of the sect. *Hedysarum* [33,35]. Besides, in karyotypes of the species from groups 2, 3, and 4, additional 5S and 45S rDNA clusters were revealed on other chromosome pairs, which can be related to chromosome rearrangements as well as intra- and interspecific hybridization events occurred during speciation.

The sect. *Hedysarum* is the widest-ranging section within the genus *Hedysarum*. Plant species belonging to this section are distributed in temperate to boreal regions of the Northern Hemisphere and occur in various habitats such as alpine and arctic meadows, stone grasslands, deserts and seashores [4,16]. The constructed here integrated schematic map allowed us to specify the species occurrence and analyze spatial overlap in species distributions within Eurasia. In the regions where populations of related species grow together, introgressive hybridization events can occur. Several new species having hybrid background were earlier detected within the *Hedysarum* sect. *Multicaulia* [42,43]. At the same time, according to the constructed map, *H. flavescens* and *H. caucasicum* have rather narrow distribution ranges. These species normally grow in high mountain areas [16,17,18,40]. Most probably, the both species are relics of former distribution areas, which were probably much wider, and now are isolated from the other members having large overlapping areas in boreal and arctic zones.

*H. alpinum*, *H. hedysaroides*, *H. arcticum,* and *H. neglectum* are the most common species of the sect. *Hedysarum* [16,36]. Among them, *H. alpinum* covers the widest ranges of habitats including Europe, Siberia, the Far East, China, the Korean Peninsula and northern parts of Mongolia [36]. *H. theinum* is an endemic species growing in the Altai Mountains [36,37]. The distribution area of this species overlaps (partly or almost totally) with the ranges of *H. hedysaroides*, *H. arcticum*, *H. austrosibiricum*, and *H. neglectum.* These species can occupy different ecological niches, in particular, *H. theinum* grows in the subalpine and, rarely, alpine meadows, whereas *H. neglectum* occurs in the forest zone [36,37,44]. Nonetheless, there are still problems with the correct plant identification, especially in the regions with multiple species occurrence. This can be related to hybridization and introgression events as well as morphological similarities observed in closely related species of this section [2,4,6]. The chromosome analysis with the use of cytogenomic markers could specify some closely related species of the sect. *Hedysarum*.

The systematic positions of *H. arcticum*, *H. hedysaroides*, and *H. austrosibiricum* are still uncertain due to their high morphological similarities. In different reports, *H. arcticum* was described as an independent plant species [16,36] or a subspecies of *H. hedysaroides* [6,45]. Our results indicate that *H. arcticum* and *H. hedysaroides* have overlapping geographical areas. According to recent phylogenetic cp data, *H. hedysaroides* and *H. arcticum* are grouped in one clade [33,35].

*H. austrosibiricum* was earlier classified as a subspecies of *H. hedysaroides* [6], and currently, *H. austrosibiricum* is considered an independent species because of its narrow mountain range confined to Southern Siberia [36]. In the present study, in karyotypes of *H. hedysaroides*, we detected heteromorphic combinations of homologues chromosomes bearing 45S rDNA (NOR-I and NOR-II variants). At the same time, in karyotypes of *H. arcticum* and *H. austrosibiricum*, we revealed a single variant (different for each species) of chromosomes bearing 45S rDNA. These observations might suggest that they are actually independent species.

*H. flavescens* and also other yellow-flowering species of the sect. *Hedysarum* are considered as a primary mesophilic group distributed in highlands of the Pamir-Alay and Tien Shan Mountains. It is suggested that a part of these species could spread across Eurasia and create centers of secondary speciation, in particular, in Siberia [17,18]. The geographical distribution of *H. flavescens* mostly corresponds to the distribution areas (mountainous regions of Central Asia) of the species forming basal clades within the sect. *Hedysarum*. However, *H. flavescens* was not examined in the previous phylogenetic studies [33,34,35]. This suggests that Mountains of Central Asia could be the ancestral area for the species from this section. Currently, *H. flavescens* has a narrow distribution area in the Pamir-Alay and Tien Shan Mountains. However, this species belongs to the group of other species of the sect. *Hedysarum* (*H. arcticum*, *H. austrosibiricum*, *H. hedysaroides,* and *H. theinum*) having similar karyotypes and distributed in different regions of Eurasia. Therefore, it cannot be excluded that the genome of *H. flavescens* might be the closest to the ancestor genome.

*H. theinum* is known to be morphologically different from closely related *H. neglectum* and considered an independent species [44]. The constructed integrated schematic map of the species distribution illustrates that ranges of *H. neglectum* and *H. theinum* overlap. However, *H. theinum* is an endemic species grown in the highlands (1300–2000 m above sea level). *H. neglectum* occurs a wider range of environmental conditions (mostly, in the forest zone) compared to *H. theinum* [36,37]. These species also differ considerably from each other in chemical composition as well as amount of biologically active substances in their leaves and roots [9,44]. According to our results, *H. theinum* and *H. neglectum* have some differences in their karyotype structure. In *H. neglectum*, we detected two pairs of satellite chromosomes though only one pair was revealed in *H. theinum*, which might suggest that they were different species.

Moreover, in *H. neglectum*, we observed a number of polymorphic minor 45S rDNA sites localized on chromosome pairs 1, 2, 3, and 7, and the chromosome localization of these minor 45S rDNA loci was specific for accessions grown in different locations. In karyotypes of *H. theinum*, minor 45S rDNA loci were detected only on chromosome pairs 2. These minor loci were also polymorphic; their presence and chromosome localization varied depending on the growing area (e.g., Kazakhstan or the Altai Mountains). Our results are consistent with data on ISSR analyses which demonstrated increased intraspecific variability in *H. theinum* [30] and a higher level of interpopulation variability of the electrophoretic spectra of seed polypeptides in *H. neglectum* compared to *H. theinum* [31].

In the present study, the revealed variability in chromosome localization of minor rDNA clusters probably indicates that active processes of 45S rDNA redistribution still occur in the genomes of *H. neglectum* and *H. theinum*. High recombination rate and also increased activity of mobile elements facilitate changes in number of rDNA sites [46,47,48,49]. It was earlier assumed that the variations in number of 45S rDNA clusters observed in karyotypes of different species of *Anacyclus* could result from interspecific hybridization events occurring in the regions where ranges of these species overlapped or along the edge of their distribution areas [50]. Also, variations in the number of 45S rDNA loci were observed in karyotypes of plant species (*Phaseolus vulgaris*, *Capsicum* species, *Brassica rapa*) grown in different locations [51,52,53]. On the other hand, no variations in number and location of 45S rDNA clusters were earlier revealed among accessions of *Hedysarum perrauderianum* from different regions of Algeria [26].

In both *H. caucasicum* and *H. neglectum*, one of two pairs of satellite chromosomes was presented by a variant of chromosome 5. In karyotypes of *H. neglectum*, the other satellite chromosome (7) was detected only in this species and can be used as a species-specific chromosome marker. In karyotype of *H. caucasicum*, the other satellite chromosome pair (3) bearing both 5S and 45S rDNA clusters is also a unique variant which can be used as an additional chromosome marker for identification of this species. *H. caucasicum* is known to occupy a narrow isolated area [40], and this fact has apparently helped to maintain such a unique variant of a satellite chromosome pair in its karyotype.

As mention above, *H. alpinum* and *H. neglectum* have overlapping areas. However, these species differed in localization of 45S and 5S rDNA clusters. Our results on chromosome analysis are consistent with the previous phylogenetic data indicating that *H. alpinum* and *H. neglectum* belong to separate clades [33,35]. *H. alpinum* holds a special position among the studied species, as it is considered to be a conserved type for the genus *Hedysarum* [4]. In this species, all examined accessions had the similar karyotypes. Moreover, we did not reveal any differences in chromosomal localization of 45S and 5S rDNA clusters between the introduced and wild populations. At the same time, in karyotypes of *H. alpinum*, we detected two chromosome pairs bearing 5S rDNA clusters unlike the other species of this section having one chromosome pair with 5S rDNA. The exception was one accession of *H. hedysaroides* (K 445-17) which also had two chromosome pairs bearing 5S rDNA. It cannot be excluded, however, that this accession has been misidentified by a taxonomist, and in fact, it also belongs to *H. alpinum*. It is therefore possible that the presence of the second chromosome pair (4) bearing 5S rDNA in a karyotype can be used as additional species-specific character in taxonomic studies of the sect. *Hedysarum.*

## 4. Materials and Methods

### 4.1. Plant Material

In the present study, we examined twenty plant accessions covering eight *Hedysarum* species obtained from different seed sources (detailed in Table 1) including the collection of All-Russian Institute of Medicinal and Aromatic Plants (AIMAP), Moscow, RF. Wild plant accessions were collected and identified by Dr. I. Yu. Selyutina and Dr. N.A. Karnaukhova, the Central Siberian Botanical Garden (CSBG), SB RAS, RF and Dr. N.A. Suprun, Volgograd Regional Botanical Garden (VRBG), Volgograd, RF.

### 4.2. Chromosome Spread Preparation

Plant seeds were scarified and put into hot (60–75 °C) water for 5–10 min. Then they were germinated in Petri dishes for 3–7 days at 22 °C. For cell cycle synchronization and accumulation of mitotic divisions, excised root tips (of 0.5 cm long) were incubated in ice water for 24 h. After this pre-treatment, the root tips were fixed in ethanol:acetic acid (3:1) for 3–24 h at room temperature. Before squashing, the roots were transferred into 1% acetocarmine solution in 45% acetic acid for 15 min. The cover slips were removed after freezing in liquid nitrogen. The slides were dehydrated in 96% ethanol and then air dried.

### 4.3. FISH Procedure

For a FISH procedure, following probes were used: pTa71 which comprised a 9 kb long DNA sequence of common wheat including 18S, 5.8S and 26S (45S) rDNA [54]; pTa794 which comprised a 420 bp long DNA sequence of wheat including 5S rDNA [55]. These DNA probes were labelled directly with fluorochromes SpectrumAqua or SpectrumRed by nick translation according to manufacturers’ protocols (Abbott Molecular, Wiesbaden, Germany). The FISH procedure was carried out as described previously [56]. After hybridization, the slides were washed respectively in 0.1 × SSC at 50 °C for 5 min, 2 × SSC at 37 °C for 10 min, 2 × SSC at RT for 5 min and 1 × PBS at RT for 3 min. Then the slides were dehydrated through a graded ethanol series, air dried and stained with 0.1 μg/mL DAPI (4′,6-diamidino-2-phenylindole) (Serva, Heidelberg, Germany) in Vectashield mounting medium (Vector laboratories, Peterborough, UK).

### 4.4. Chromosome Analysis

At least five plants of each accession and fifteen metaphase plates from each sample were analyzed. The slides were examined using an Olympus BX-61 epifluorescence microscope (Olympus, Tokyo, Japan). Images were captured with a monochrome charge-coupled device camera (Cool Snap, Roper Scientific, Inc., Sarasota, FL, USA). Then they were processed with Adobe Photoshop 10.0 software (Adobe, Birmingham, AL, USA).

## 5. Conclusions

For eight *Hedysarum* species, we constructed the integrated schematic map of their distribution within Eurasia based on currently available scattered data. For the first time, we performed cytogenomic characterization of twenty accessions covering eight closely related species from the sect. *Hedysarum*. Intra- and interspecific variability of chromosomes bearing 45S and 5S rDNA clusters was detected. Based on chromosome analysis, we revealed four main karyotype groups in the studied species: (1) *H. arcticum*, *H. austrosibiricum*, *H. flavescens*, *H. hedysaroides,* and *H. theinum*; (2) *H. alpinum* and one accession K 445-17 of *H. hedysaroides*; (3) *H. caucasicum;* and (4) *H. neglectum*. *H. caucasicum*, *H. neglectum,* additional cytogenomic markers were observed. The detected species-specific chromosomal markers can be useful in taxonomic studies of this section.

## Figures and Tables

**Table 1:** The list of the studied plant accessions.

Species	Accession Number/Voucher	Origin/Seed Source
*H. alpinum* L.	HA-O-1964	Omsk region, RF/germplasm collection of CSBG, 2009
*H. alpinum* L.	K 04-08	Unknown/germplasm collection of AIMAP, 2017
*H. alpinum* L.	ZUD_29082007	51°7′ N; 112°11′ E; Zabaikalye region, RF/collected by Dr. N.A. Karnaukhova, 2007
*H. alpinum* L.	BBB_18082017	53°58′ N; 113°35′ E; Bauntovskii region, Buryatia, RF/collected by Dr. I.Yu. Selyutina, 2017
*H. arcticum* B. Fedtsch.	K 02252/818_24072016	67°25′ N; 65°11′ E; lake Shchuch’e, Komi Republic, RF/collected by Dr. N.A. Suprun, 2016
*H. arcticum* B. Fedtsch.	K 499-17	VRBG/germplasm collection of AIMAP, 2017
*H. arcticum* B. Fedtsch.	K 292-19	VRBG/germplasm collection of AIMAP, 2017
*H. austrosibiricum* B. Fedtsch.	KZ_RIP_25071998	50°19′ N; 83°50′ E; Ivanovskiy Ridge, 2000 m above sea level, Kazakhstan/collected by Dr. I.Yu. Selyutina, 1998
*H. caucasicum* M. Bieb.	K 202-2018	Gothenburg Botanical Garden, Sweden/germplasm collection of AIMAP, 2018
*H. flavescens* Rgl. et Schmalh.	CSBG_Fl_U30072007	University of Uppsala Botanical Garden, Sweden, Uppsala/germplasm collection of CSBG, 2008
*H. hedysaroides* (L.) Schinz et Thell.	K 518-17	Botanical Garden of the University of Vienna, Austria/germplasm collection of AIMAP, 2017
*H. hedysaroides* (L.) Schinz et Thell.	K 445-17	University of Tartu Botanical Gardens, Estonia/germplasm collection of AIMAP, 2017
*H. hedysaroides* (L.) Schinz et Thell.	K 96-19	Botanical Garden of the University of Vienna, Austria/germplasm collection of AIMAP, 2017
*H. neglectum* Ledeb.	K 4/4-99	Kaira Yalbak Mountains, Altai region, RF/germplasm collection of AIMAP, 2017
*H. neglectum* Ledeb.	K 692-02	Altai region/germplasm collection of AIMAP, 2017
*H. neglectum* Ledeb.	AUK_26082004	50°39′ N; 87°43′ E; Kubadru River, Altai region, RF/collected by Dr. I.Yu. Selyutina, 2004
*H. neglectum* Ledeb.	AUKYA_28072008	50°52′ N; 85°15′ E; Yabogansky pass, Altai region, RF/collected by Dr. N.A. Karnaukhova, 2008
*H. theinum* Krasnob.	AUKK_15082008	50°59′ N; 84°13′ E; Kumir river, Altai region, RF/collected by Dr. I.Yu. Selyutina, 2008
*H. theinum* Krasnob.	KZ_RP_25072008	50°18′ N; 83°38′ E; Ridderskii region, Kazakhstan/collected by Dr. I.Yu. Selyutina, 2008
*H. theinum* Krasnob.	AUSK_05092008	50°4′ N; 85°13′ E; Ust-Koksinsky region; Altai Mountains, RF/collected by Dr. N.A. Karnaukhova, 2008

## Data Availability

All data are contained within the article.
